# Prevalence, diversity of diarrhoeagenic *Escherichia coli* and associated risk factors in well water  in Ile-Ife, Southwestern Nigeria

**DOI:** 10.1186/s42522-021-00057-4

**Published:** 2022-02-08

**Authors:** Babatunde Odetoyin, Olawumi Ogundipe, Adebola Onanuga

**Affiliations:** 1 Department of Medical Microbiology and Parasitology, College of Health Sciences, Obafemi Awolowo University, Ile-Ife, Nigeria; 2 Department of Pharmaceutical Microbiology and Biotechnology, Faculty of Pharmacy, University of Maiduguri, Maiduguri, Borno State Nigeria

**Keywords:** *Escherichia coli*, Diarrhoea, Well water, Risk factors, Diversity, Contamination, Prevalence

## Abstract

**Background:**

Diarrhoeagenic *Escherichia coli* (DEC) strains are common causes of morbidity and mortality worldwide. Waterborne DEC could pose a health risk to humans through domestic use of contaminated water. However, epidemiological studies on DEC in well water are scarce in Nigeria. This study determined the prevalence, diversity and factors associated with the presence of DEC in well water in Ile-Ife, southwestern Nigeria.

**Methods:**

We assessed 143 wells for safety and a questionnaire was administered. Contaminating isolates were identified as *E. coli* by amplifying their *16S rRNA* gene. Five diarrhoeagenic *E. coli* pathotypes were sought using multiplex polymerase chain reaction (PCR). (GTG)5 repetitive PCR and Shannon diversity index were used to determine isolates diversity. Multivariate analysis was used to reveal the factors associated with the presence of DEC in  well water.

**Results:**

Fifty-six (39.2%) wells were contaminated by diarrhoeagenic *E. coli*. Wells with dirty platforms, undercut by erosion and sited near septic tanks significantly harboured DEC (*p* <  0.05). There was a preponderance of Shiga-toxin producing *E. coli* among the isolates with 10 (17.9%) wells contaminated by multiple DEC. The DEC isolates showed 45 unique fingerprints and were divided into six clades, with an overall diversity index of 18.87.

**Discussion:**

The presence of DEC in well water highlights the risk to human health associated with the use of untreated water. There was a high degree of genetic diversity among the isolates implying multiple sources of contamination. There is a need for periodic sanitation and inspection of wells for cracks to prevent seepages and possible outbreaks of waterborne diseases.

## Background

Diarrhoeal diseases are significant public health problems in developing countries [1]. Each year, they account for 3.6% of the total global burden of diseases and 1.5 million deaths. About 88% of this burden has been ascribed to inadequate hygiene, sanitation and a lack of potable water mostly in developing countries [1, 2]. *Escherichia coli,* a member of faecal coliforms has a significant place in water microbiology as an indicator of faecal pollution and a pathogen in drinking water. As a pathogen, it causes a variety of diseases ranging from urinary tract infections, sepsis, meningitis and bacteraemia to diarrhoea [3].

Diarrhoeagenic *Escherichia coli* (DEC) account for about 40% of episodes of acute diarrhoea in children in developing countries. They also play a significant causative role in diarrhoea in Nigeria, in both adults and children. Currently, there are eight pathotypes of DEC strains: enterotoxigenic, enterohaemorrhagic, enteroinvasive, enteropathogenic, enteroaggregative, diffusely adherent, cytolethal distending toxin-producing and cell detaching *E. coli*. Each pathotype of DEC has a distinct set of virulence factors encoded in the plasmids or chromosome. The genes that encode these factors are conserved among strains that are isolated from diverse sources in different parts of the world [4].

DEC strains are usually transmitted via a faecal-oral route which involves contaminated sources of water or food and may be involved in outbreaks of waterborne diarrhoea. *Escherichia coli* can enter drinking water via inadequate or failing septic or sewer systems, runoff from land applied with animal wastes or animal feeding operations and wildlife. Identification of the source of pollution is a high priority in order to protect source water quality and to assess the public health risk associated with contamination from a particular host source. Consequently, much progress has been made over the years to develop many phenotypic and genotypic microbial source tracking (MST) methods which are recommended components of faecal pollution reduction strategies [5, 6].

Nigeria is one of the countries in the world where about 90 million people don’t have access to potable water and 130,000 children under the age of five die each year from avertable waterborne diseases due to uncoordinated efforts of various agencies of government. The larger part of the population, particularly those in the rural and suburban communities resort to water from wells and streams for domestic purposes [2, 7]. Those wells which are hand dug are usually around 4–15 ft in diameter and about 25 ft deep. In Ile-Ife, most of the wells are shallow because of the high water table. Shallow wells are more prone to contamination due to their proximity to the soil surface and potential source of contamination [8, 9]. These alternative sources of water are largely untreated and might harbour waterborne pathogens. Therefore, the use of these sources of water is a health risk for this population [7, 10].

Despite the risk posed by exposure to *E. coli* contaminated water, very little data is available on this in Ile-Ife, and the pathogenic potential, diversity of implicated isolates and factors associated with their presence in well water remain unknown. Therefore this study determined the prevalence, diversity and factors associated with the presence of DEC in well water in Ile-Ife, Southwestern Nigeria.

## Methods

### Study location and design

The study was done in Ife East Local Government Area, Ile-Ife, Osun State, Nigeria. Ife East Local Government Area is divided into six wards which are: Moore ward, Ilode ward 1, Ilode ward 2, Okerewe ward 1, Okerewe ward 2 and Okerewe ward 3. Ile-Ife is an ancient city in southwestern Nigeria with a population of 509, 035 [11]. The city lies on Latitudes 7°28′N and 7°45′N and longitudes 4°30′E and 4°34′E. Ile-Ife is in the tropical wet and dry climate of West Africa with an average rainfall of 1000 to 1250 mm between March and October and average relative humidity of 75 to 100%.

### Study approval and sample collection

This study was approved by the Health Research Ethics Committee (HREC), Institute of Public Health, Obafemi Awolowo University, Ile-Ife, Nigeria (HREC No: IPHOAU/12/863). A total of 143 water samples were collected from wells that are distributed across the wards between March and December 2019 based on the formula of Sullivan and Soe [12]. The wells are used by the residents for domestic purposes. Wells that have not been disinfected for two months were included in the study while wells of owners that did not give their consent, and those that were disinfected were excluded. Up to 200 ml of water were obtained by lowering a sterile bottle into each well with the aid of a rope tied around its neck. All the samples were labelled appropriately, placed in an ice-packed box and transported within 2 h to the laboratory for processing.

### Determination of well water quality

The quality of the samples was determined using the multiple tube fermentation technique as described by Cheesbrough [13]. A three-tube most probable number (MPN) method was used to determine faecal contamination of well water using MacConkey broth (Oxoid Ltd., Basingstoke Hampshire, England) as the culture medium. Samples of 50 ml, 10 ml and 1 ml of water were inoculated into corresponding dilution tubes with inverted Durham’s tubes and incubated at 37 °C for 24 h. The tubes were observed for growth and gas production, and the MPN of coliforms in 100 ml of water was determined by referring to McCrady’s table and interpreted as “Excellent”, “Acceptable”, “Unacceptable” and “Grossly polluted”.

### Detection of *Escherichia coli* in water samples

The Eijkman method was used to detect the presence of *E. coli* in the samples. All positive bottles from the previous test were subcultured into fresh double strength and single strength MacConkey broth and peptone water and incubated at 37 °C for 24 h. The MacConkey bottles were checked after incubation for lactose fermentation (yellow colouration) and gas production (presence of a bubble in the Durham tubes). All positive MacConkey bottles were noted and three drops of Kovac’s reagent were added to their corresponding peptone water bottles to detect indole (indicated by a red coloured ring). All positive samples were cultured on Eosin Methylene Blue Agar plates and incubated aerobically at 37 °C for 24 h. Up to three distinct colonies showing green metallic sheen were aseptically picked and streaked onto Nutrient agar (NA) (Oxoid Ltd., Basingstoke Hampshire, England) plates which were, in turn, incubated aerobically at 37 °C for 24 h [14]. All suspected *E. coli* isolates were stored at -20 °C in glycerol broths for further examination.

### Isolate resuscitation and DNA extraction

All isolates were subcultured from glycerol broths on nutrient agar plates and incubated at 37 °C for 24 h. Three colonies were picked from each culture and suspended in 50μl of sterile distilled water in an Eppendorf tube to extract the DNA of the isolates. The suspension was boiled for 10 min, kept on ice for 10 min, and centrifuged at 10,000 rpm for 10 min [15]. The supernatant was collected and used as a DNA template in PCR reactions.

### Molecular identification of isolates by amplifying their gene

All organisms suspected to be *E. coli* by their phenotypic characteristics were confirmed as *E. coli* by amplifying their *16S rRNA* gene (Table 1) [16]. *E. coli* strain 25922 was used as the positive control while water was used as the negative control. A 25 μl reaction mixture contained 12.5μL of 2XMaster mix, 10 pmol each of the primers (Eurofins, USA), 2.4 μl of the DNA template and made up with Nuclease Free Water. Amplification conditions were as follows: Initial denaturation at 95 °C for 5 min; 35 cycles of denaturation at 94 °C for 45 s, annealing at 45 °C for 45 s, and extension at 72 °C for 1 min; followed by a final extension at 72 °C for 5 min. Each PCR product (10 μl) was electrophoresed on a 1.5% (w/v) agarose gel in 1X TAE. Gels containing 5ul of 10μg/ml of ethidium bromide were visualized under ultraviolet (UV) light using a UVitec transilluminator (Avebury, Cambridge UK).

**Table 1:** PCR primers for diarrhoeagenic *Escherichia coli* and *16srRNA* gene

Type	Primer Designation	Primers (5 to 3)	Target gene	Amplicon size (bp)
**ECO**	ECO-1	GACCTCGGTTTAGTTCACAGA	*16SrRNA*	585
	ECO-2	CACACGCTGACGCTGACCA		
**EPEC**	eae 1	CTGAACGGCGATTACGCGAA	*eae*	917
	eae 2	CCAGACGATACGATCCAG		
	bfp 1	AATGGGCTTGCGCTTCCAG	*bfpA*	326
	bfp 2	GCCGCTTTATCCAACCTGGTA		
**EAEC**	EAEC1	CTGGCGAAAGACTGTATCAT	*CVD432*	630
	EAEC2	CAATGTATAGAAATCCGCTGTT		
**ETEC**	LTf	GGCGACAGATTATACCGTGC	*LT*	450
	LTr	CAATGTATAGAAATCCGCTGTT		
	STf	ATTTTTMTTTCTGTATTRTCTT	*ST*	190
	STr	CACCCGGTACARGCAGGATT		
**EIEC**	IpaH1	GTTCCTTGACCGCCTTTCCGATACCGTC	*ipaH*	600
	IpaH2	GCCGGTCAGCCACCCTCTGAGAGTAC		
**EHEC**	Stx1f	ATAAATCGCCATTCGTTGACTAC	*Stx1*	180
	Stx1r	AGAACGCCCACTGAGATCATCC		
	Stx2f	GGCACTGTCTGAAACTGCTCC	*Stx2*	255
	Stx2r	TCGCCAGTTATCTGACATTCTG		

*EIEC* Enteroinvasive *E. coli, EHEC* Enterohemorrhagic *E. coli, EAEC* Enteroaggregative *E. coli, EPEC* Enteropathogenic *E. coli, ETEC* Enterotoxigenic *E. coli*

### Detection of diarrhoeagenic genes in the isolates

All isolates were screened for virulence genes characteristic of five pathotypes of diarrhoeagenic *E. coli* comprising enteroinvasive *E. coli* (EIEC), enteropathogenic *E. coli* (EPEC), enterotoxigenic *E. coli* (ETEC), enteroaggregative *E. coli* (EAEC) and enterohaemorrghagic *E. coli* (EHEC) including shiga toxin producing *E. coli* (STEC) as described by Aranda et al. [17] with modifications (Table 1). PCR was performed with a 20 μl reaction mixture containing 12.5uL 2XMaster mix, 10 pmol each of PCR primers (Eurofins, USA), 2.4 μl of the DNA template and made up with Nuclease Free Water. Two PCR reaction assays were used to amplify the eaeA (intimin of EHEC and EPEC), *bfpA* (bundle-forming pilus of EPEC), *stx1* and/or *stx2* (shiga toxins 1 and 2 of EHEC and STEC), *eltB* and/or *estA* (enterotoxins LT and ST of ETEC), *ipaH* (invasion plasmid found in EIEC and *Shigella*) and pCVD (pCVD432 of EAEC). *E. coli* strains E2348/69, O42, H10407, EDL 933 and E137 served as positive controls for EPEC, EAEC, ETEC, EHEC and EIEC respectively while sterile water was used as a negative control. For PCR 1 (eae, CVD432, stx1, ipaH, ST): Amplification conditions were as follows: Initial denaturation at 95 °C for 3 mins; 37 cycles of denaturation at 94 °C for 30 s, annealing at 45 °C for 30 s, and extension at 72 °C for 1 min; followed by a final extension at 72 °C for 7 min. For PCR 2 (stx2, bfp, LT): Amplification conditions were as follows: Initial denaturation at 94 °C for 3 mins; 37 cycles of denaturation at 94 °C for 45 s, annealing at 39 °C for 30 s, and extension at 72 °C for 54 min; followed by a final extension at 72 °C for 7 min. Each PCR product (10 μl) was electrophoresed on a 1.5% (w/v) agarose gel in 1X TAE. Gels containing 5ul of 10μg/ml of ethidium bromide were visualized under ultraviolet (UV) light using a UVitec transilluminator (Avebury, Cambridge UK).

### Determination of isolates relatedness and diversity

(GTG) 5-PCR was used to subtype the isolates. PCR was performed with a 25 μl reaction mixture containing 12.5uL 2XMaster mix, 10 pmol each of the primer (5’GTGGTGGTGGTGGTG3’), 2.4 μl of the DNA template and made up with Nuclease Free Water. Amplification conditions were as follows: Initial denaturation at 95 °C for 5 mins; 35 cycles of denaturation at 95 °C for 60 s, annealing at 40 °C for 60 s, and extension at 68 °C for 8 min; followed by a final extension at 68 °C for 8 min. Each PCR product (10 μl) was electrophoresed on a 1.5% (w/v) agarose gel in 1X TAE [18]. Gels containing 5ul of 10μg/ml of ethidium bromide were visualized under ultraviolet (UV) light using a UVitec transilluminator (Avebury, Cambridge UK). GelJ (Version 1.0) software was used to generate isolates similarity index [19]. The dendrogram was drawn with PAST (Version 4.0) software using neighbour-joining clustering method [20].

The genetic diversity of DEC isolates was calculated using the Shannon diversity index (*H*) formula [21].

$${H}=-\sum \limits_{i=1}^S{p}_i\ln p{}_i$$

*i* is the total number of isolates, *s* is the number of unique genotypes and *pi* is the number of isolates sharing the same genotype.

### Data analysis

Data analysis was done with R statistical software (Version 4.0.3). Cross tables were produced with *the Grammar of Tables* in R package. Pearson chi-square and binomial logistic regression models were used to test for association of variables with the presence of DEC in water [22]. The *P*-value for a significant association was set at 0.05.

## Results

### Characteristics of wells

**Fig. 1:**
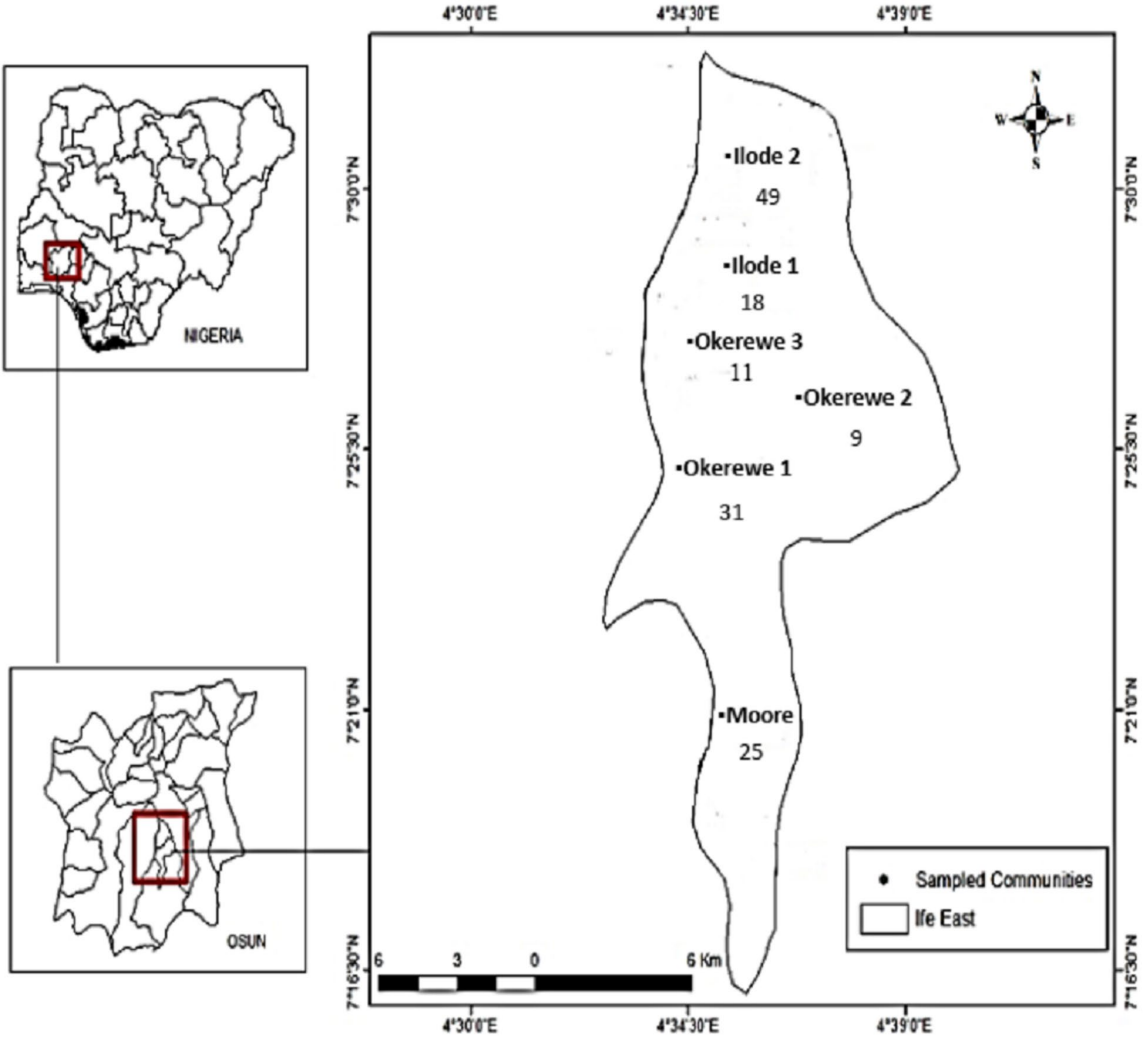
Sampled locations and Number of wells (*n* = 143)

This study investigated water quality and characterized *Escherichia coli* in the study area from 143 wells. The sampling locations are shown on the map in Fig. 1 Twenty-five samples were obtained from Moore ward, 18 samples from Ilode ward 1, 49 samples from Ilode ward 2, 31 samples from okerewe ward 1, 9 samples from okerewe ward 2 and 11 samples from okerewe ward 3 (Table 2). Most of the wells were covered (*n* = 108; 75.5%), some were partially covered (*n* = 20; 13.99%), and a few were not covered (*n* = 15; 10.5%). The majority of well owners were Christians (111, 78.7%), artisans (100, 69.9%) with secondary education (63, 50%) and lived in tenement (81, 56.6%). The mean age of the wells was 21 years and the average depth was 29.3 ft.

**Table 2:** Baseline characteristics of wells and owners

Characteristics	Overall (***N*** = 143)
**Wards**	
Ilode 1	18 (12.6%)
Ilode 2	49 (34.3%)
Moore	25 (17.5%)
Okerewe 1	31 (21.7%)
Okerewe 2	9 (6.3%)
Okerewe 3	11 (7.7%)
**Age of wells (Mean** ± SD; years)	20.6 ± 21.7
**Depth of wells (Mean** ± SD; Feet)	29.3 ± 22.1
**Mean of age (Mean** ± SD; years)	45.8 ± 17
**Mean of number of years in residence (Mean** ± SD; years)	14.2 ± 16.4
**Religion**	
Christianity	111 (78.7%)
Islam	26 (18.4%)
Traditionalist	4 (2.8%)
**Occupation**	
Artisan	100 (69.9%)
Civil servant	28 (19.6%)
Religious leader	5 (3.5%)
Student	6 (4.2%)
Unemployed	4 (2.8%)
**Level of education**	
Primary	24 (19.0%)
Secondary	63 (50.0%)
Tertiary	39 (31.0%)
**Residence type**	
Flat	62 (43.4%)
Tenement	81 (56.6%)
**Covered**	
Covered	108 (75.5%)
Open	15 (10.5%)
Partially covered	20 (14.0%)
**Presence of septic tank**	
No	94/140 (67.1%)
Yes	46/140 (32.9%)
**Keeping of pets**	
No	96/138 (69.6%)
Yes	42/138 (30.4%)
**Dirty platform**	
No	104 (72.7%)
Yes	39 (27.3%)
**Proximity of livestock to well**	
No	102 (71.3%)
Yes	41 (28.7%)
**Proximity of waste dump site to well**	
No	137 (95.8%)
Yes	6 (4.2%)
**Proximity of well to farm**	
No	133 (93.0%)
Yes	10 (7.0%)
**Well undercut by erosion**	
No	116 (81.1%)
Yes	27 (18.9%)

### Contaminated wells and isolated *Escherichia coli* strains

One hundred and ten (110, 76.9%) wells were contaminated with coliforms bacteria. Ilode ward 2 (36; 32.7%) had the highest number of contaminated wells while Okerewe ward 3 (6; 5.5%) had the least number (Table 3).

**Table 3:** Isolates distribution in the wards in the local government

Wards	Locations	Number of wells	Number of wells contaminated with coliform bacteria	***E. coli*** Isolated	No of wells with ***E. coli***
**Moore**	Moore	6	4	7	4
	Opa	5	4	3	2
	Iloromu	1	0	0	0
	Mokuro	12	11	17	8
	Olopo	1	1	3	1
**Subtotal**	**5**	**25**	**20**	**30**	**15**
**Ilode 1**	Oke atan	7	7	12	5
	Lokore	10	9	6	4
	Ayelabowo	1	1	1	1
**Subtotal**	**3**	**18**	**17**	**19**	**10**
**Ilode 2**	Oke ogbo	31	22	27	17
	Omitoto	7	5	10	5
	Ogooluwatan	10	9	19	8
**Subtotal**	**3**	**49**	**36**	**56**	**30**
**Okerewe 1**	Iloro	3	0	0	0
	Okesoda	5	4	5	4
	Ayetoro	16	13	21	12
	Oke ayetoro	3	3	3	2
	Gbodo	4	4	8	3
**Subtotal**	**5**	**31**	**24**	**37**	**21**
**Okerewe 2**	Ita agbon	1	1	1	1
	Otutu	2	2	4	2
	Ajamopo	2	2	2	1
	Lakanye	2	2	5	2
	Itakogun	1	0	0	0
**Subtotal**	**5**	**9**	**7**	**12**	**6**
**Okerewe 3**	Ogbonya	11	6	15	8
**Subtotal**	1	11	6	15	8
**Total**	**22**	**143**	**110**	**169**	**98**

A total of 169 *E. coli* strains were isolated from 98 wells of 110 contaminated wells. As shown in Table 3, 30 strains were isolated from the wells in Moore ward, 19 strains from Ilode ward1, 56 strains from Ilode ward 2, 37 strains from Okerewe ward 1, 12 strains from Okerewe ward2 and 15 strains from Okerewe ward 3.

### Prevalence of Diarrhoeagenic *Escherichia coli*

**Fig. 2:**
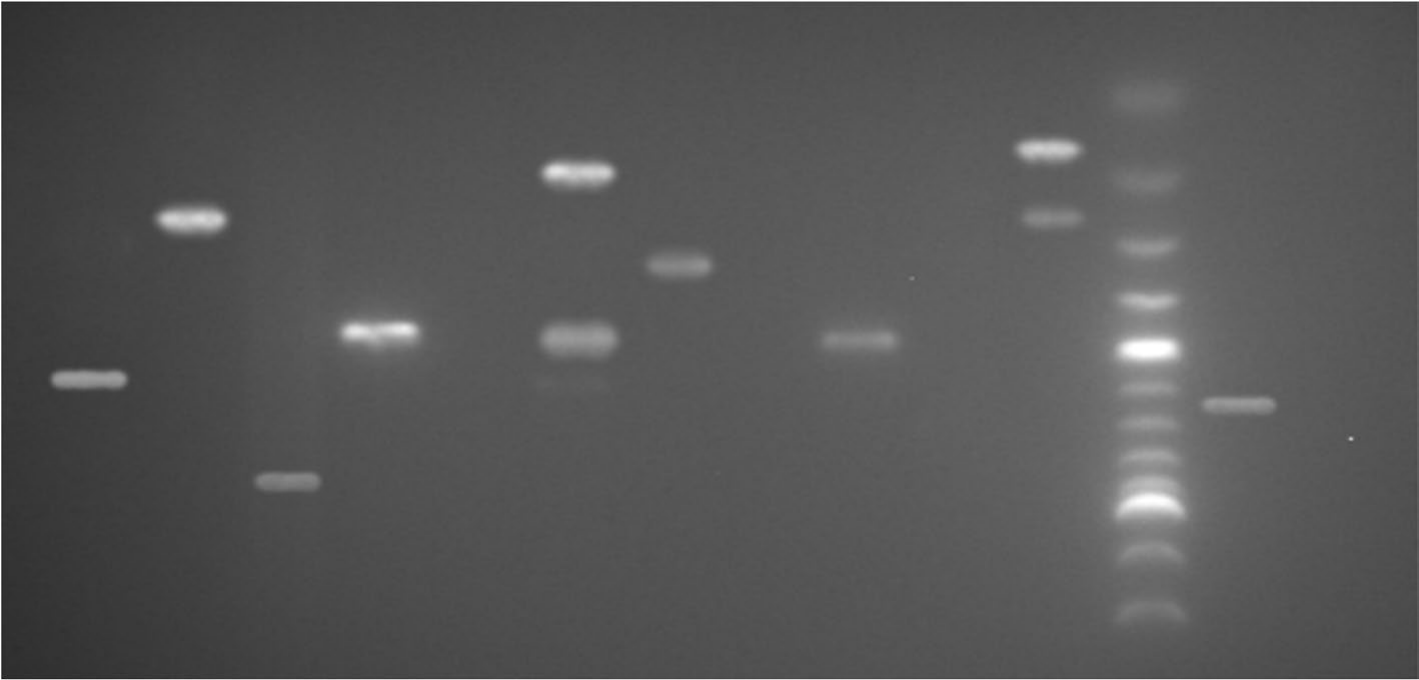
A representative gel picture showing diarrhoeagenic virulence genes of water isolates. Lane 1: Water (Negative); Lane 2: E. coli 042 (CVD432-630bp); Lane L: 100bp ladder; Lane 3: E. coli EDL 933 (stx1-180bp, stx2-255bp); Lane 4: E. coli ; Lane 5: E. coli H10407 (LT-450bp); Lane 6: E. coli; Lane 7: E. coli E2348 (bfp-326bp); Lane 8: E. coli H10407 (ST-190bp and LT-450bp): Lane 9: E. coli; Lane 10: E. coli (LT-450bp); Lane 11: E. coli (eae-917bp); Lane 12: E. coli (stx2-255bp); Lane 13: E. coli E137 (ipaH-600bp)

Two sets of PCR assays were used to determine the prevalence of eight distinct virulence genes possessed by five *E. coli* pathotypes. Up to three strains of *E. coli* were isolated from each water sample and examined for diarrhoeagenic genes. The detailed results of the analysis are in Fig. 2, Tables 4 and 5.

**Table 4:** Number of samples collected, positive for *E. coli* and positive for diarrhoeagenic *E. coli*

**Locations**	Total sampled wells	No of *E. coli* Isolated	No of wells with DEC	DEC Isolates	EAEC	ETEC	EIEC	STEC	EHEC	EPEC	STEC AND tEPEC	ETEC AND STEC	tEPEC, ETEC AND STEC	EAEC, tEPEC
**Moore**	25	30	12	15	0	1	0	12	0	2	0	0	0	0
**Ilode1**	18	19	7	7	0	0	0	4	1	0	1	1	0	0
**Ilode 2**	49	56	12	14	0	0	1	9	0	2	1	1	0	0
**Okerewe 1**	31	37	15	16	0	6	2	1	2	2	0	3	0	0
**Okerewe 2**	9	12	5	9	1	2	0	5	0	0	0	0	1	0
**Okerewe 3**	11	15	5	8	0	1	1	4	0	1	0	0	0	1
**Total**	143	169	56	69	1	10	4	35	3	7	2	5	1	1

*EIEC* Enteroinvasive *E.coli, EHEC* Enterohemorrhagic *E. coli, EAEC* Enteroaggregative *E. coli, EPEC* Enteropathogenic *E. coli, ETEC* Enterotoxigenic *E. coli, STEC* Shiga toxin producing *Escherichia coli, tEPEC* typical Enteropathogenic *E. coli*

**Table 5:** Number and type of DEC isolated from sampled locations

S/N	Strain number	Pathotype	Genes	Locations	Wards
1	111a	EHEC	*Stx2 + Eae*	Ayelabola	Ilode 1
2	92w	STEC AND tEPEC	*Stx2 + Bfp*	Lokore	Ilode 1
3	Ds85cii	ETEC AND STEC	*ST + Stx2*	Lokore	Ilode 1
4	Ds94dii	STEC	*Stx2*	Okeatan	Ilode 1
5	Ds96cii	STEC	*Stx2*	Oke Atan	Ilode 1
6	Ds97dii	STEC	*Stx2*	Oke Atan	Ilode 1
7	Ds99eii	STEC	*Stx2*	Oke Atan	Ilode 1
8	13bw	STEC	*Stx1*	Omitoto	Ilode 2
9	18aw	STEC	*Stx2*	Oke Ogbo	Ilode 2
10	37wi	STEC AND tEPEC	*Stx2 + Bfp*	Ogooluwatan	Ilode 2
11	64ssbi	STEC	*Stx2*	Oke Ogbo	Ilode 2
12	6ew	STEC	*Stx2*	Ogooluwatan	Ilode 2
13	7350 ml	STEC	*Stx2*	Omitoto	Ilode 2
14	7b	STEC	*Stx2*	Ogooluwatan	Ilode 2
15	Ds50c	STEC	*Stx2*	Oke Ogbo	Ilode 2
16	Ds65aii	ETEC AND STEC	*ST + Stx2*	Oke Ogbo	Ilode 2
17	Ds73e	tEPEC	*Bfp*	Omitoto	Ilode 2
18	Ds76aiii	STEC	*Stx2*	Ogooluwatan	Ilode 2
19	Ds79ci	EIEC	*Ipah*	Ogooluwatan	Ilode 2
20	Ds80a	tEPEC	*Bfp*	Ogooluwatan	Ilode 2
21	Ds80aiii	STEC	*Stx1*	Ogooluwatan	Ilode 2
22	115	tEPEC	*Bfp*	Mokuro	Moore
23	117	STEC	*Stx2*	Mokuro	Moore
24	126	STEC	*Stx1*	Olopo	Moore
25	108a	STEC	*Stx2*	Mokuro	Moore
26	109a	ETEC	*ST*	Moore	Moore
27	109b	STEC	*Stx2*	Mokuro	Moore
28	114c	STEC	*Stx1*	Mokuro	Moore
29	116a	STEC	*Stx2*	Mokuro	Moore
30	119b	STEC	*Stx2*	Opa	Moore
31	123a	STEC	*Stx1*	Mokuro	Moore
32	123b	STEC	*Stx2*	Mokuro	Moore
33	126c	STEC	*Stx2*	Olopo	Moore
34	23cwii	STEC	*Stx2*	Opa	Moore
35	4aw	tEPEC	*Bfp*	Moore	Moore
36	Ds122a	STEC	*Stx2*	Mokuro	Moore
37	124c	ETEC AND STEC	*ST + Stx2*	Gbodo	Okerewe 1
38	125a	EHEC	*Stx2 + Eae*	Gbodo	Okerewe 1
39	130c	EHEC	*Stx1 + Eae*	Ayetoro	Okerewe 1
40	131b	tEPEC	*Bfp*	Ayetoro	Okerewe 1
41	132b	ETEC AND STEC	*ST + Stx2*	Oke Soda	Okerewe 1
42	138b	tEPEC	*Bfp*	Ayetoro	Okerewe 1
43	139b	ETEC	*ST*	Ayetoro	Okerewe 1
44	142a	ETEC	*ST*	Ayetoro	Okerewe 1
45	142di	ETEC	*LT*	Ayetoro	Okerewe 1
46	143c	ETEC	*ST*	Oke Soda	Okerewe 1
47	154a	EIEC	*Ipah*	Ayetoro	Okerewe 1
48	154b	STEC	*Stx2*	Ayetoro	Okerewe 1
49	69wii	ETEC AND STEC	*ST + Stx2*	Ayetoro	Okerewe 1
50	Ss145eii	ETEC	*ST*	Oke Ayetoro	Okerewe 1
51	142diii	ETEC	*ST*	Ayetoro	Okerewe 1
52	Ds144ciii	EIEC	*Ipah*	Oke Ayetoro	Okerewe 1
53	107a	ETEC	*ST*	Lakanye	Okerewe 2
54	127a	STEC	*Stx1*	Otutu	Okerewe 2
55	127b	STEC	*Stx2*	Otutu	Okerewe 2
56	128a	ETEC	*ST*	Otutu	Okerewe 2
57	128b	tEPEC, ETEC AND STEC	*Bfp + St + Stx2*	Otutu	Okerewe 2
58	128c	STEC	*Stx2*	Otutu	Okerewe 2
59	148a	STEC	*Stx2*	Ajamopo	Okerewe 2
60	150b	STEC	*Stx1*	Itakogun	Okerewe 2
61	Ds42c	EAEC	*Cvd432*	Itakogun	Okerewe 2
62	101a	STEC	*Stx1*	Ogbonya	Okerewe 3
63	101b	EIEC	*Ipah*	Ogbonya	Okerewe 3
64	102b	tEPEC	*Bfp*	Ogbonya	Okerewe 3
65	103b	STEC	*Stx2*	Ogbonya	Okerewe 3
66	105a	ETEC	*ST*	Ogbonya	Okerewe 3
67	105b	STEC	*Stx2*	Ogbonya	Okerewe 3
68	152a	STEC	*Stx2*	Ogbonya	Okerewe 3
69	152b	EPEC, EAEC	*Cvd432 + Bfp*	Ogbonya	Okerewe 3

Fifty-six (39.2%) wells were contaminated by diarrhoeagenic *E. coli* (DEC), yielding a total of 69 DEC strains. Okerewe 1(*n* = 15) had the highest number of wells that were contaminated with DEC, while Okerewe 3(*n* = 5) had the least number. There was a preponderance of STEC (*n* = 35) among the strains, followed by ETEC (*n* = 10). Two and five strains were both STEC/tEPEC and ETEC/STEC respectively. Multiple pathotypes of DEC were recovered from 10 (17.9%) wells.

### Factors associated with DEC contamination of wells

Of the wells that were contaminated by DEC, 16 (28.6%) were undercut by erosion, 26 (46.4%) were sited near septic tanks, 24(41.4%) had dirty platforms, 22 (37.9%) were owned by those who keep pets, 39(69.6%) were used by those in a tenement, 19(33.9%) were sited near livestock and 40(71.4%) were owned by artisans. The average age and depth of the wells were 17.5 ± 22.2 (mean ± SD; Years) and 31.5 ± 23.5 (mean ± SD; Feet) respectively (Table 6).

**Table 6:** Univariate analysis of risk factors for contamination with DEC

Characteristics	No (***N*** = 87)	Yes (***N*** = 56)	Total (***N*** = 143)	***p***-value
***Wards***				0.183^a^
**Ilode 1**	11.0 (12.6%)	7.0 (12.5%)	18.0 (12.6%)	
**Ilode 2**	37.0 (42.5%)	12.0 (21.4%)	49.0 (34.3%)	
**Moore**	13.0 (14.9%)	12.0 (21.4%)	25.0 (17.5%)	
**Okerewe 1**	16.0 (18.4%)	15.0 (26.8%)	31.0 (21.7%)	
**Okerewe 2**	4.0 (4.6%)	5.0 (8.9%)	9.0 (6.3%)	
**Okerewe 3**	6.0 (6.9%)	5.0 (8.9%)	11.0 (7.7%)	
***Age of well owners (Mean ± SD; years)***	44.3 ± 16.3	48.1 ± 17.9	45.8 ± 17	0.200^b^
***Number of years in resisdence (Mean ± SD; years)***	16.58 ± 18.6	12.7 ± 14.8	14.2 ± 16.4	0.168^b^
***Age of wells (Mean ± SD; years)***	25.4 ± 20.2	17.5 ± 22.2	20.6 ± 21.7	0.033^b^
***Depth of wells (Mean ± SD; Feet)***	25.8 ± 19.5	31.5 ± 23.5	29.3 ± 22.1	0.128^b^
***Well undercut by erosion***				0.018^a^
**No**	76.0 (87.4%)	40.0 (71.4%)	116.0 (81.1%)	
**Yes**	11.0 (12.6%)	16.0 (28.6%)	27.0 (18.9%)	
***Gender***				0.053^a^
**Female**	70.0 (80.5%)	37.0 (66.1%)	107.0 (74.8%)	
**Male**	17.0 (19.5%)	19.0 (33.9%)	36.0 (25.2%)	
***Religion***				0.621^a^
**Christianity**	69.0 (80.2%)	42.0 (76.4%)	111.0 (78.7%)	
**Islam**	14.0 (16.3%)	12.0 (21.8%)	26.0 (18.4%)	
**Traditionalist**	3.0 (3.5%)	1.0 (1.8%)	4.0 (2.8%)	
***Level of education***				0.334^a^
**Primary**	16.0 (20.5%)	8.0 (16.7%)	24.0 (19.0%)	
**Secondary**	35.0 (44.9%)	28.0 (58.3%)	63.0 (50.0%)	
**Tertiary**	27.0 (34.6%)	12.0 (25.0%)	39.0 (31.0%)	
***Covered***				0.227^a^
**Covered**	70.0 (80.5%)	38.0 (67.9%)	108.0 (75.5%)	
**Open**	7.0 (8.0%)	8.0 (14.3%)	15.0 (10.5%)	
**Partially covered**	10.0 (11.5%)	10.0 (17.9%)	20.0 (14.0%)	
***Presence of septic tank***				0.005^a^
**No**	64.0 (76.2%)	30.0 (53.6%)	94.0 (67.1%)	
**Yes**	20.0 (23.8%)	26.0 (46.4%)	46.0 (32.9%)	
***Keeping of pets***				0.035^a^
**No**	64.0 (76.2%)	32.0 (59.3%)	96.0 (69.6%)	
**Yes**	20.0 (23.8%)	22.0 (40.7%)	42.0 (30.4%)	
***Proximity of livestock to well***				0.265^a^
**No**	65.0 (74.7%)	37.0 (66.1%)	102.0 (71.3%)	
**Yes**	22.0 (25.3%)	19.0 (33.9%)	41.0 (28.7%)	
***Proximity of waste dump site to well***				0.578^a^
**No**	84.0 (96.6%)	53.0 (94.6%)	137.0 (95.8%)	
**Yes**	3.0 (3.4%)	3.0 (5.4%)	6.0 (4.2%)	
***Proximity of well to farm***				0.198^a^
**No**	79.0 (90.8%)	54.0 (96.4%)	133.0 (93.0%)	
**Yes**	8.0 (9.2%)	2.0 (3.6%)	10.0 (7.0%)	
***Residence type***				0.012^a^
**Flat**	45.0 (51.7%)	17.0 (30.4%)	62.0 (43.4%)	
**Tenement**	42.0 (48.3%)	39.0 (69.6%)	81.0 (56.6%)	
***Occupation***				0.131^a^
**Artisan**	60.0 (69.0%)	40.0 (71.4%)	100.0 (69.9%)	
**Civil servant**	18.0 (20.7%)	10.0 (17.9%)	28.0 (19.6%)	
**Religious leader**	2.0 (2.3%)	3.0 (5.4%)	5.0 (3.5%)	
**Student**	6.0 (6.9%)	0.0 (0.0%)	6.0 (4.2%)	
**Unemployed**	1.0 (1.1%)	3.0 (5.4%)	4.0 (2.8%)	
***Dirty platform***				< 0.001^a^
**No**	72.0 (82.8%)	32.0 (57.1%)	104.0 (72.7%)	
**Yes**	15.0 (17.2%)	24.0 (42.9%)	39.0 (27.3%)	
***Hospitalization in last year***				0.542^a^
**No**	67.0 (83.8%)	43.0 (79.6%)	110.0 (82.1%)	
**Yes**	13.0 (16.2%)	11.0 (20.4%)	24.0 (17.9%)	
***Marital status***				0.045^a^
**Married**	75.0 (86.2%)	54.0 (96.4%)	129.0 (90.2%)	
**Single**	12.0 (13.8%)	2.0 (3.6%)	14.0 (9.8%)	

^a^Pearson chi-square test; ^b^Student t test

Univariate analysis revealed that wells that were undercut by erosion (*p* = 0.018), sited near septic tanks (0.005), had dirty platforms (0.001), owned by those who kept pets (0.035), used by those in tenement (0.012) significantly harboured diarrhoeagenic *E*.*coli*.

The associated factors were further subjected to multivariate analysis using the binomial logistic regression model. Wells that were undercut by erosion (OR = 2.616, CI = 1.019–6.716, *p* = 0.046), sited near septic tank (OR = 2.611, CI = 1.131–6.027, *p* = 0.025), had dirty platforms (OR = 3.125, CI = 1.232–7.924, *p* = 0.016) were significantly associated with the presence of DEC in wells. However, there was no significant association between wells that were owned by those who kept pets (OR = 0.884, CI = 0.335–2.329, *p* = 0.803) and those used in tenement (OR = 1.115, CI = 0.418–2.977, *p* = 0.828) and the presence of diarrhoeagenic *E. coli* (Table 7).

**Table 7:** Multivariate Logistic regression models of DEC in the assessed wells

Predictor			Odds ratio	Lower	Upper	*P*-value
Well undercut by erosion	Yes	16.0 (28.6%)				
	No	11.0 (12.6%)	2.616	1.019	6.716	0.046
Presence of septic tank	Yes	26.0 (46.4%)				
	No	20.0 (23.8%)	2.611	1.131	6.027	0.025
Dirty platform	Yes	24.0 (42.9%)				
	No	15.0 (17.2%)	3.125	1.232	7.924	0.016
Keeping of pets	Yes	22.0 (40.7%)				
	No	20.0 (23.8%)	0.884	0.335	2.329	0.803
Residence type	Tenement	39.0 (69.6%)				
	Flat	42.0 (48.3%)	1.115	0.418	2.977	0.828

### Relatedness and diversity of DEC isolates

**Fig. 3:**
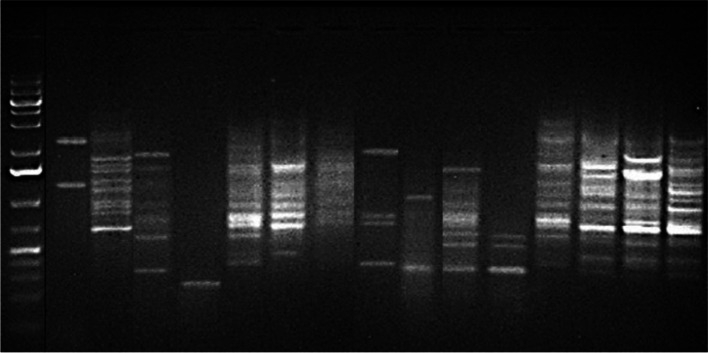
A representative picture of (GTG)5 PCR fingerprints of DEC isolates

**Fig. 4:**
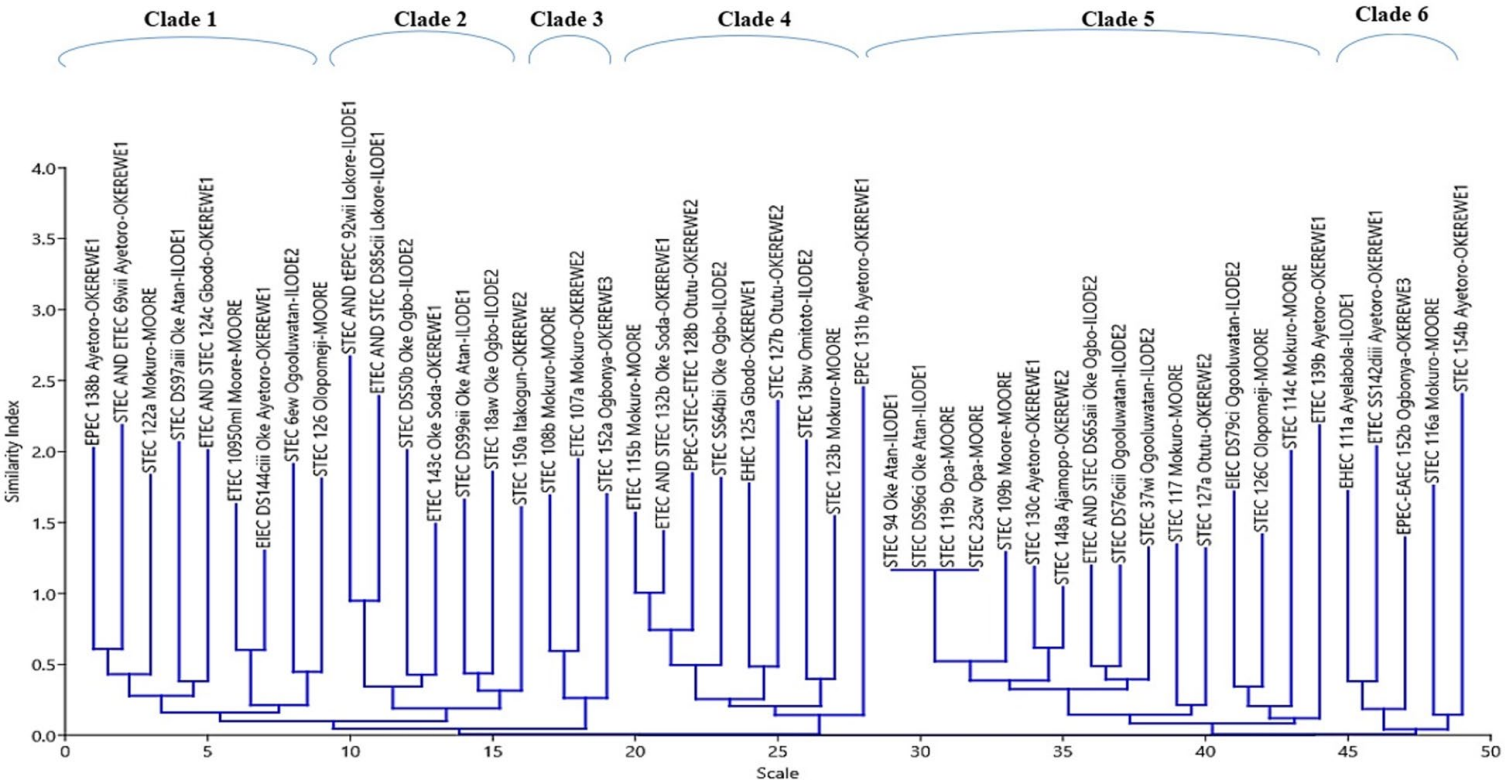
Neighbour-joining dendrogram clusters of (GTG)5-fingerprints of strains with their locations

Repetitive PCR was used to determine the relatedness of the DEC isolates. A representative (GTG)5-PCR fingerprint picture is shown in Fig. 3. Isolates banding patterns ranged from 1 to 14 bands. Bands molecular weight varied from 100 bp to 4706 bp. Fifty DEC isolates were typed by (GTG)5 while certain isolates did not produce any band and appeared not typeable. The (GTG)5-PCR fingerprints dendrogram is shown in Fig. 4. All the isolates clustered together. Nevertheless, six clades of strains were observed along the axis from 0 to 45. Clade 5 had the highest number of strains (12/50; 24%), while clade 3 had the least number (3/50; 6%). Four STEC isolates (119b-Opa-Moore, 23cw-Opa-Moore, 96-Oke Atan-Ilode1 and 94-Oke Atan- Ilode 1) from different locations and wards in the local government in Clade 5 are identical.

In all, the isolates were highly diverse as indicated by Shannon diversity index (H = 18.87). Isolates from Okerewe ward 1 (H = 5.41) were the most diverse while those from Okerewe ward 3 were the least diverse (H = 3.17). Other diversity indices are: Moore (H = 4.93), Ilode ward 1 (H = 4.68), Ilode ward 2 (H = 4.93) and Okerewe ward 2 (H = 4.60).

## Discussion

Diarrhoeal disease is a leading cause of morbidity and mortality in children globally and a high percentage of bacterial gastroenteritis is caused by diarrhoeagenic *E. coli* (DEC) [1]. In Nigeria, epidemiological studies on DEC isolates in drinking water are scarce. To the best of our knowledge, this is the first study in Nigeria that will investigate the presence of DEC in well water.

In this study, 169 *E. coli* strains were isolated from 98 out of 110 wells that were contaminated by coliform bacteria. All the isolates were screened for eight different diarrhoeagenic genes possessed by five *E. coli* pathotypes. We detected DEC in 56 wells in the six wards of the local government area. Our observation aligns with the reports of previous investigators which observed that drinking water can be a reservoir of DEC in the environment [23, 24]. The prevalence of DEC in our study (39.2%) is relatively higher than that of da Silva et al. [25] (28.1%) and Taomaneso et al. [23] (33.3%), but similar to 48% reported by Ali et al. [4] The prevalence of DEC pathotypes appears to vary according to geographical region probably due to different prevailing risk factors. Largely, the presence of potentially pathogenic *E. coli* in drinking water highlights the potential risk for environmental transmissibility of these strains in different parts of the world.

In order to identify the risk factors associated with the presence of DEC in water in the study environment, we used binomial logistic regression models to test for association. Our analysis revealed a significant association between the presence of DEC and wells that were undercut by erosion, sited near septic tanks and those with dirty platforms. Findings from previous studies have also highlighted these factors to have a significant association with water contamination [26–29]. Siting of septic tanks close to wells could result in leakages or seepages of faecal material into the wells thereby contaminating groundwater. This was evident in a USA study that assessed the seasonal correlation of septic tank distance and well contamination and found a significant connection between decreasing distance and increasing coliform between septic tanks and wells [30]. Similarly, a review of pit latrines and their impacts on groundwater quality by Graham et al. (2013) concluded that in order to avoid groundwater contamination, latrines and water sources should be at least 50 m apart [31]. Also, cracks in the wells can expose wells to polluted storm water and agricultural runoffs. Hence, the knowledge of associated risk factors can provide information that can generate ideas for workable interventions.

We observed that the DEC pathotypes’ prevalence varied according to location, probably due to the prevailing associated factors in each location. Okerewe ward 1 had the highest number of wells that were contaminated with DEC, while Okerewe 3 had the least number. Furthermore, multiple DEC pathotypes were recovered from ten wells in the sampled locations. Previous studies in Burkina Faso [32], Bangladesh [33] and Brazil [34] have reported similar findings, implying multiple sources of contamination of the wells.

All the five pathotypes of DEC that we sought were identified with a preponderance of STEC. The occurrence of STEC in drinking water has been reported globally [34, 35]; along with outbreaks of waterborne disease caused by this pathotype [36, 37]. STEC are public health concerns due to their ability to cause anaemia, uraemia and kidney failure, especially in young children. Our observation is in tandem with previous studies that had detected STEC in drinking water [35, 38]. Our prevalence is higher than that of Elmonir et al. [24] in Egypt (33.3%). In contrast, none of the *E. coli* isolates from water samples in France was STEC [39]. Interestingly, our previous study on the prevalence of DEC in diarrheic children in this environment also showed a preponderance of STEC amongst other pathotypes that were identified [15]. Therefore, this study indicates that STEC is prevalent in this environment and water could be a  reservoir.

Most of our STEC harboured *stx2* which is strongly associated with haemorrhagic colitis and haemolytic uraemic syndrome in humans. Even though *eae* is a significant determinant of virulence in STEC infection, most of the *stx2* -positive isolates did not have it, apart from three isolates that harboured *eae* with *stx2* and *stx*_*1*_. While considering the reported health risk attributable to STEC, the detection of *eae*-negative STEC strains in our study could be a public health concern as outbreaks of bloody diarrhoea and hemolytic-uremic syndrome (HUS) caused by STEC strains without the *eae* gene have been reported, which suggests that Shiga toxin is the primary virulence trait responsible for HUS [34, 36]. Besides, the *stx2* gene has been documented to be more strongly associated with severe disease in humans than the *stx1*, thus, signifying its importance in human infection.

ETEC, EAEC, EPEC have been linked with waterborne outbreaks of gastroenteritis. In our study, ETEC was second to STEC in terms of prevalence. Kambire et al. [40] found that 90% of *E. coli* isolated from water were ETEC which differs from the prevalence of 14.5% we got in our study, but higher than Rodrigues da Silva et al. [25] that reported less than 1%. EAEC strains have been linked with outbreaks of gastroenteritis in South Korea due to consumption of contaminated groundwater [36]. In this study, EAEC was the least prevalent pathotype. Also, a study conducted in South Africa, showed that only EAEC was found of all the DEC strains sought [41]. The EPEC strains are of two types; atypical EPEC (aEPEC) and typical EPEC (tEPEC). Humans are the only reservoir for tEPEC, which is spread by inter-human contact. Canizalez-Roman et al. [42] and Sidhu et al. [43] detected tEPEC in food and surface water respectively. The detection of only tEPEC in our study suggests that the wells were contaminated by humans. Also, the detection of EPEC as the third most prevalent pathotypes in our study shows that contaminated water can be a source of infection by this pathotype in humans.

EIEC is an important *E. coli* pathotype that causes watery diarrhoea and dysentery similar to *Shigella* in terms of pathogenesis. In this study, EIEC was detected in four (5.8%) DEC isolates. Compared with our findings, higher prevalence rates of EIEC have been reported from China (9.1%) [44] and Sudan (41.3%) [45] probably due to geographical differences.

Moreover, our results showed two and three combinations of diarrhoeagenic genes of different *E. coli* pathotypes isolated from some water samples: STEC and tEPEC (*N* = 2/56) (3.6%), ETEC and STEC (*N* = 5/56) (8.9%), tEPEC, ETEC and STEC (1/56)(1.8%), EAEC and tEPEC (1/56) (1.8%). Remarkably, this is the first study to report these combinations in waterborne DEC isolates. Other studies reported a different combination of genes from both EAEC and EHEC [43, 46]. This finding is of a public health concern as mixed infections usually involve more dehydration compared with episodes caused by a single DEC pathotype.

There have been reports on the prevalence of DEC pathotypes in healthy and diseased individuals from Nigeria; however, there is a paucity of waterborne DEC studies that reveal the relatedness of isolates according to their sources of isolation. Therefore, to determine the degree of diversity among DEC pathotypes, all isolates were subjected to (GTG)5 rep-PCR typing, a genotypic technique for the detection of diversity. In our study, complex fingerprint patterns were obtained for all DEC isolates. In addition, all the DEC isolates clustered together with six clades of strains observed. Generally, we obtained a diverse profile among and between the isolates recovered from different sources. The highly adaptive nature of *E. coli* with a short generation time interval as well as easy acquisition of mobile genetic elements under selection pressure provides a greater degree of genetic diversity among *E. coli* strains. The extensive diversity among *the DEC* strains isolated from different sources largely rules out between/within location transmissibility of isolates. Likewise, several independent studies have reported the existence of diverse populations of *E. coli* in several hosts and environments [5, 47]. Clade 5 had the highest number of strains (12/50; 24%), while clade 3 had the least number (3/50; 6%). Four STEC isolates from different locations and wards in the local government in Clade 5 were identical. This implies that these isolates have either been maintained or circulated within a similar source of origin. Our isolates were highly diverse as indicated by the Shannon diversity index (H = 18.87). The diversity of isolates implies multiple sources of contamination at the locations.

## Conclusions

This study reports a high prevalence of DEC in well water with a preponderance of STEC. The presence of these pathogenic strains of *E. coli* in drinking water highlights the risk to human health associated with the use of untreated water. There was a high degree of genetic diversity among the isolates implying multiple sources of contamination thus emphasizing the need for periodic sanitation and inspection of wells for cracks to prevent seepages, runoff and possible outbreaks of waterborne diseases. Also, there is a need to sensitise well owners and consumers to inculcate the habit of boiling untreated water before use. Regulatory agencies in charge of well construction and water quality must take the appropriate measures to ensure proper well siting, construction, and maintenance to prevent contamination.

## Data Availability

All data and materials of this study are included. If additional information is needed, please contact the author for requests.

## Abbreviations

DEC: Diarrhoeagenic *Escherichia coli*
EPEC: Enteropathogenic *Escherichia coli*
EHEC: Enterohaemorrhagic *Escherichia coli*
EIEC: Enteroinvasive *Escherichia coli*
ETEC: Enterotoxigenic *Escherichia coli*
STEC: Shiga toxin producing *Escherichia coli*
MPN: Most probable number
ATCC: American type culture collection
